# Unified Protocol for the transdiagnostic treatment of emotional disorders in people with post COVID-19 condition: study protocol for a multiple baseline n-of-1 trial

**DOI:** 10.3389/fpsyg.2023.1160692

**Published:** 2023-10-18

**Authors:** Verónica Martínez-Borba, Laura Martínez-García, Óscar Peris-Baquero, Jorge Osma, Esther del Corral-Beamonte

**Affiliations:** ^1^Institute for Health Research Aragón (IIS Aragón), Zaragoza, Spain; ^2^Universidad de Zaragoza, Zaragoza, Spain; ^3^Villanova Royo Hospital, Zaragoza, Spain

**Keywords:** post COVID-19, long COVID-19, Unified Protocol, transdiagnostic, psychological intervention, multiple baseline, study protocol

## Abstract

**Background:**

Post COVID-19 syndrome, defined as the persistence of COVID-19 symptoms beyond 3 months, is associated with a high emotional burden. Post COVID-19 patients frequently present comorbid anxiety, depressive and related disorders (emotional disorders, EDs) which have an important impact on their quality of life. Unfortunately, psychological interventions to manage these EDs are rarely provided to post COVID-19 patients. Also importantly, most psychological interventions do not address comorbidity, namely simultaneous EDs present in COVID-19 patients. This study will explore the clinical utility and acceptability of a protocol-based cognitive-behavioral therapy called the Unified Protocol for the transdiagnostic treatment of EDs in patients suffering post COVID-19 condition.

**Methods:**

A multiple baseline n-of-1 trial will be used, as it allows participants to be their own comparison control. Sample will be composed of 60 patients diagnosed with post COVID-19 conditions and comorbid EDs from three Spanish hospitals. After meeting the eligibility criteria, participants will answer the pre-assessment protocol and then they will be randomly assigned to three different baseline conditions (6, 8, or 10 days of assessments before the intervention). Participants and professionals will be unblinded to participants’ allocation. Once the baseline assessment has been completed, participants will receive the online psychological individual intervention through video-calls. The Unified Protocol intervention will comprise 8 sessions of a 1 h duration each. After the intervention, participants will answer the post-assessment protocol. Additional follow-up assessments will be conducted at one, three, six, and twelve months after the intervention. Primary outcomes will be anxiety and depressive symptoms. Secondary outcomes include quality of life, emotion dysregulation, distress tolerance, and satisfaction with the programme. Data analyses will include between-group and within-group differences and visual analysis of patients’ progress.

**Discussion:**

Results from this study will be disseminated in scientific journals. These findings may help to provide valuable information in the implementation of psychological interventions for patients suffering post COVID-19 conditions.

**Clinical trial registration:**

https://clinicaltrials.gov, identifier (NCT05581277).

## 1. Introduction

In December 2019, the COVID-19 disease was originated in Wuhan (China) and currently it is considered one of the greatest pandemics ever. According to the data reported by the World Health Organization (WHO), it is estimated that 657 millions of people worldwide have contracted the infection and more than 6 millions of people have died, which has had an impact in 220 countries (WHO, 2022a).

The symptoms more frequently reported in patients with COVID-19 infections include, but are not limited to, fever, cough, tiredness, loss of taste or smell, sore throat, headache, and shortness of breath (WHO, 2021). While most COVID-19 patients recover from these symptoms, it is also well-known that an important proportion of patients get no relieve from COVID-19 symptoms, a condition known as post COVID-19 condition or long COVID-19. By definition, patients who continue to experience or develop new symptoms of COVID-19 three months after contracting COVID-19 are diagnosed as long COVID patients (WHO, 2022b). Nowadays, it is difficult to determine the precise prevalence of post COVID-19 conditions in COVID-19 survivors, but estimations establish that around 10–20% of people infected by the virus develop long COVID-19 condition (WHO, 2022b).

Aside from the physical consequences, the pandemics itself and the subsequent restriction measures (i.e., lockdown, reduction of social contact, job instability, and economic difficulties) have had an impact on the psychological wellbeing of the general population (Liu et al., 2021). A recent meta-analysis with 146,139 participants from nine different countries found that the most common symptoms during the COVID-19 crisis were emotional disorders, namely anxiety, depressive and related disorders (hereafter EDs; Barlow et al., 2018). This meta-analytic study found high prevalence rates of anxiety (32.60%), insomnia (30.30%), depression (27.60%) and post-traumatic stress disorder (16.70%) with increased prevalence of EDs in patients with suspected and confirmed COVID-19 infection (63.90% reported anxiety symptoms and 55.40% depressive symptoms; Liu et al., 2021).

In the particular case of patients suffering post COVID-19 conditions, the most frequently reported psychological problems were also anxiety (22.20%) and depressive disorders (21.10%; Deer et al., 2021). Apart from this, patients suffering long COVID conditions may be at increased risk of ED chronification as they may have to cope not only with physical symptoms but also with loneliness and the stigma related to scepticism about their symptoms, which may in turn compromise their social and family life, work functionality and quality of life (Brown and O’Brien, 2021; Office of National Statistics, 2021).

In light of the aforementioned psychological negative consequences of the pandemic, it might be reasonable to think that patients with COVID-19, and especially those with long COVID-19, would receive evidence-based psychological interventions to overcome EDs. Different systematic reviews indicated that psychological interventions are available in the context of COVID-19 for the general population and students (D’Onofrio et al., 2022; Komariah et al., 2022), professionals and informal caregivers (Bertuzzi et al., 2021), relatives (Yue et al., 2020), or even COVID-19 patients (Rahmati and Khalili, 2022). Results from these systematic reviews indicate that psychological interventions could be effective in the management of EDs in the context of COVID-19 conditions, however, some needs are not properly addressed. First, few studies are focused on specific long COVID-19 patients. To the best of our knowledge, only one study (Bogucki and Sawchuk, 2022) provided a Cognitive Processing Therapy to manage Post Traumatic Stress Disorder in a female patient with recurrent COVID-19 infections and a diagnosis of post COVID-19 condition. Second, specific symptom-based interventions are usually provided (Chennapragada et al., 2022), which makes it difficult to address comorbid EDs, for instance, anxiety and depressive symptoms that are simultaneously present in COVID-19 patients (Joli et al., 2022).

As an alternative to symptom-focused approaches, different transdiagnostic therapies have emerged during the last decades. The Unified Protocol (UP) for the transdiagnostic treatment of EDs (Barlow et al., 2018) is one example of a transdiagnostic cognitive-behavioral psychological intervention focused on training in adaptive emotion regulation skills. Contrary to previous psychological interventions, the UP is not focused on specific symptoms associated with different disorders if not on shared factors, for example neuroticism, which can explain the development and maintenance of these emotional disorders. Neuroticism has been defined as the tendency to experience unpleasant emotions (anxiety, fear, irritability, sadness, etc.) frequently and intensely in response to different sources of stress (Barlow et al., 2014), and has been shown to underlie the origin of EDs, as well as the high comorbidity present in these disorders (Brown and Barlow, 2009). Therefore, intervening on this mechanism would allow us to address through a single treatment, a wide number of disorders (Barlow et al., 2014).

Recent systematic reviews and meta-analysis evidence that the UP could help to manage EDs in the general population (Sakiris and Berle, 2019; Cassiello-Robbins et al., 2020; Carlucci et al., 2021; Longley and Gleiser, 2023) and also in individuals with comorbid medical conditions such as those living with Human Immunodeficiency Virus (HIV), cancer, chronic pain or multiple sclerosis, among others (Osma et al., 2021). Also importantly, it has been proposed that emotion regulation could act as a transdiagnostic mechanism for mental health in the context of COVID-19 (Volkert et al., 2021) thus it could be reasonable to think that training in emotion regulation skills (i.e., practicing emotion awareness, cognitive flexibility, emotion driven behaviors and expositions) may result in the improvement of clinical symptomatology (i.e., anxiety and depressive symptoms) in that patients. However, one of the main research problem is that to the best of our knowledge, no UP-based intervention has been implemented in the specific population of long COVID-19 patients.

With the aforementioned information in mind, there is no doubt that psychological interventions for the treatment of psychological sequelae derived from post COVID-19 condition are urgently needed. The present manuscript describes the study protocol of a multiple baseline n-of-1 trial that aims to study the clinical utility and acceptability of the “Unified Protocol for the Transdiagnostic Treatment of Emotional Disorders” in the treatment of EDs and/or symptoms in a sample of patients with post-COVID-19 condition. Our hypothesis is that a structured UP-based psychological intervention focused on treating emotional dysregulation will be effective in the improvement of EDs and/or symptoms in a sample of patients with post COVID-19 conditions. More precisely, we expect that all outcomes’ measures (i.e., anxiety, depression, emotion dysregulation, quality of life and distress) will significantly improve from pre- to post-assessment. Additionally, we hypothesized that these improvements will be maintained long term (follow-up at one, three, six, and twelve months after the intervention). Finally, our hypothesis is that excellent acceptability (i.e., adherence rates) and satisfaction with the contents and format of the intervention will be found.

## 2. Methods and analysis

### 2.1. Design

This protocol describes the procedures of a pilot study which uses multiple baseline single case design to explore the preliminary clinical utility and acceptability of the UP in the treatment of EDs or symptoms in a sample of patients with post COVID-19 condition. This trial has been previously registered in clinicaltrials.gov on October 14th, 2022 and updated on January 20th, 2023 (NCT05581277). The present protocol follows the SPENT checklist (SPIRIT extension for N-of-1 trials; Porcino et al., 2020) that can be consulted in Supplementary material A (SPENT checklist) and B (abstract checklist).

Multiple baseline single case design is frequently used in clinical settings to explore the effect of an intervention on a reduced group of patients (Kazdin, 2011). It is selected for this trial as no control group is needed because each participant acts as their own control comparison (Kratochwill et al., 2012). Current guidelines recommend three different baseline conditions with at least 3–5 data points in each group (Kratochwill et al., 2010, 2012). Accordingly, we will assign participants to three different baseline conditions: 6, 8 and 10 days of assessments prior to the beginning of the intervention. Participants will by randomly allocated to one of the three baseline conditions.

### 2.2. Participants

#### 2.2.1. Eligibility

This is a secondary study derived from a bigger project called “ARACOV.” Participants in our study will be composed of a subsample of the ARACOV multicentre project. The ARACOV project aims to characterize patients with long COVID-19 and assess the impact of a nutritional and rehabilitation intervention for the improvement of quality of life in a cohort of patients with post COVID-19 condition. According to the procedures of this project, participants with long COVID-19 that are interested in participating in the project ARACOV will contact recruiting nurses. In this step nurses will conduct an extensive interview to explore if patients are able to participate in this primary ARACOV project. Patients who are not included in this specialized nutritional intervention and rehabilitative treatment due to exclusion criteria (see supplementary material C) would be offered the possibility of participating in the secondary study that aims to implement a transdiagnostic psychological intervention. Eligibility criteria to participate in this transdiagnostic psychological intervention include: being long COVID-19 adult patients (over 18 years old), presenting emotional disorders or symptoms and residing in the autonomous community of Aragón (see Table 1).

**Table 1:** Eligibility criteria to participate in the present study based on the application of a psychological transdiagnostic intervention.

Inclusion	Exclusion
Enough understanding of Spanish. Documented COVID-19 infection (i.e., PCR, Ag test or serology). Presenting Post COVID-19 condition (i.e., persistence of COVID-19 symptoms beyond 12 weeks after the initial infection). Anxiety or depressive symptoms (OASIS Score equal to or greater than 8 points or ODSIS scores equal to or greater than 7 points) and/or diagnosis of emotional disorders according to ADIS-5 interview. Having Internet access. Signing informed consent.	Participating in the trial “Specialized nutritional intervention and rehabilitative treatment for improvement of quality of life in a cohort of patients with post COVID-19 condition” from the ARACOV-01 project. Symptoms were present prior to acute COVID-19 infection. Currently receiving psychological and/or pharmacological treatment for mental health issues. Having a severe mental health disorder (e.g., bipolar and related disorders, schizophrenia and related disorders, feeding and eating disorders, dissociative disorders, etc.). Active suicidal ideation at the time of first evaluation.

PCR, polymerase chain reaction; ADIS-5, anxiety and related disorders interview schedule for DSM-5; OASIS, overall anxiety severity and impairment scale; ODSIS, overall depression severity and impairment scale.

#### 2.2.2. Sample size calculation

In order to stablish the sample size required for this trial, we follow recommendations by Bell et al. (2018) which proposed that a sample size of 60 participants would be adequate considering an effect size of 0.3 and 80% of power. Following this recommendation, our sample will be comprised of 60 participants, 20 in each baseline condition (6, 8 and 10 days of assessment before the intervention).

### 2.3. Procedures

**Figure 1:**
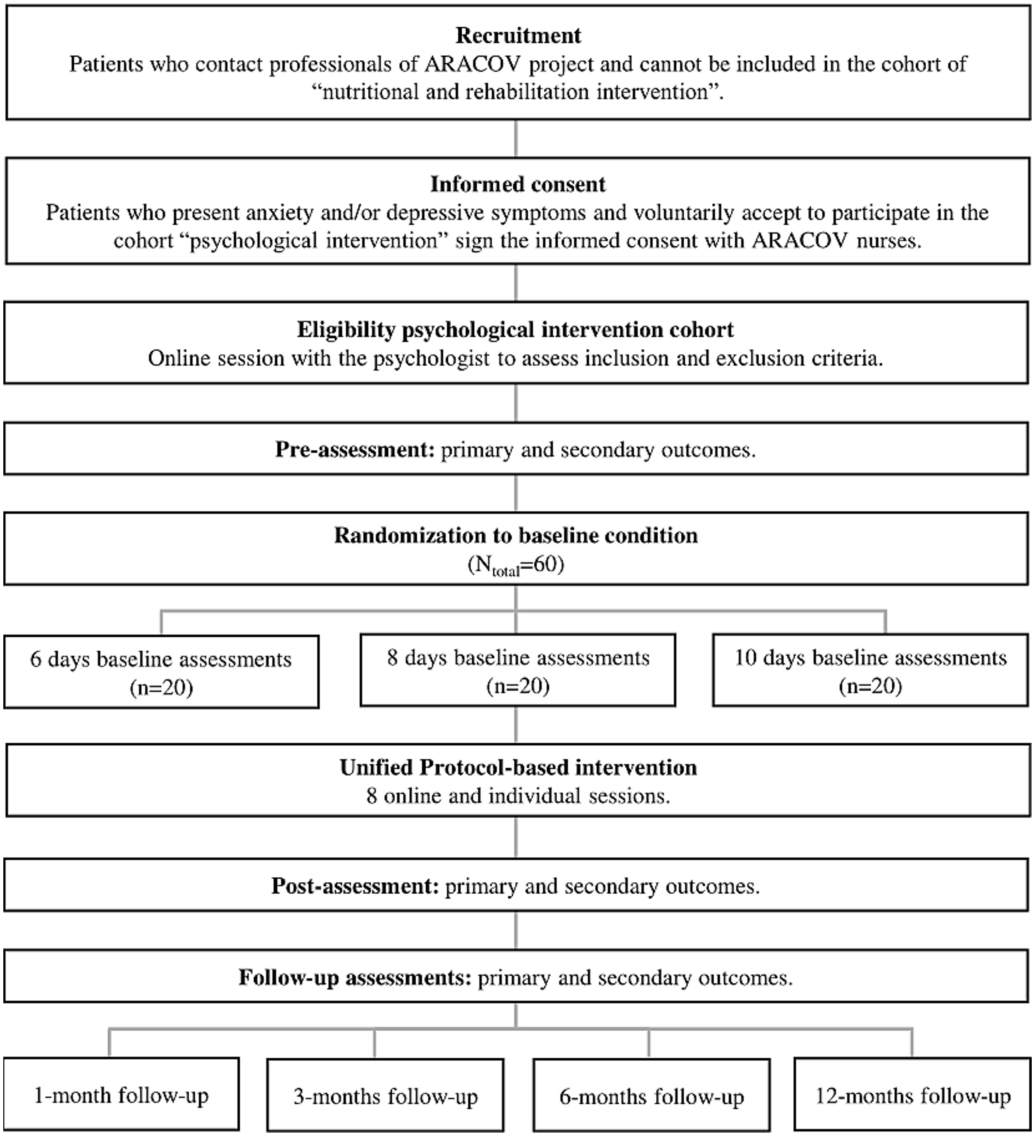
Flow chart of participants and procedures.

A complete diagram of the procedures can be consulted in Figure 1. In the present investigation, all consecutive patients presenting post COVID-19 condition and symptoms of anxiety or depression are requested to participate. Participants will be recruited by medical staff collaborating in the project ARACOV-01 in three different centres (Lozano Blesa Clinical University Hospital, Zaragoza; Huesca Pirineos Health Centre, and Teruel Ensanche Health Centre) from Spain. An additional centre, namely the Royo Villanova Hospital from Zaragoza acts as a coordinator centre (author E.D). Specifically, nurses inform patients who cannot be included in the cohort “Specialized nutritional intervention and rehabilitative treatment for improvement of quality of life in a cohort of patients with post COVID-19 condition” about the possibility of being enrolled in the present trial “Efficacy of the Unified Protocol for Transdiagnostic Treatment of Emotional Disorders in People with Post COVID-19 Condition and Emotional Symptomatology.” The principal investigator from the transdiagnostic intervention cohort is from the Health Research Institute from Aragon and Zaragoza University (J.O author).

Participants who meet the inclusion criteria (Table 1) will be given the information sheet and will sign the informed consent in the face-to-face consultation with the nursing team. Then, the psychologist of the study (V.M.B) will contact participants by telephone and will arrange an online session through a video-call to assess EDs according to the Anxiety and Related Disorders Interview Schedule for DSM-5 (ADIS-5; Brown and Barlow, 2014) which would also help to explore if any exclusion criteria are present (i.e., participants present a severe mental health issue or current suicidal ideation). Patients who meet any of these exclusion criteria will be recommended to consult with their mental health service of reference. If inclusion criteria are accomplished, participants will receive the pre-assessment protocol through e-mail and they will answer the questionnaires via the online platform Google Forms. Once the pre-assessment is completed, each participant will be sequentially and randomly assigned to one of the three multiple baseline conditions (Condition 1: 6 days of baseline assessments; Condition 2: 8 days of baseline assessments; Condition 3: 10 days of baseline assessments). During the baseline period, participants will receive daily e-mails with the online baseline assessment to be answered through Google Forms. Randomization to different baseline conditions will be conducted with a computer-generator online software (www.randomizer.org). An independent researched not involved in the implementation of the psychological intervention will sequentially assign participants to the three baseline conditions. Participants and professionals will know the baseline condition to which patients have been assigned.

After the baseline phase, participants will receive the UP-based psychological intervention. The psychologist responsible of the intervention (author VM-B) is trained in the application of the UP and will be supervised by an expert researcher which is certified by the Unified Protocol Institute as a trainer and researcher in the UP (author JO). After the intervention, 5 assessments will be made: one at post-treatment and four at different follow-ups (at one, three, six, and twelve months after completion of the intervention). A continuous monitoring of symptoms will be conducted. The psychologist responsible for the implementation of the psychological intervention could recommend that patients stop participating in the trial if aggravation of symptoms that require a specialized and more intensive psychological intervention are detected. Patients can drop out from the study at any point on request. If low adherence is detected, the psychologist will contact participants by telephone and e-mail to encourage adherence and solve problems to engage in the trial.

### 2.4. Outcomes

The assessment protocol of the study includes nine assessment points (eligibility, baseline, pre-assessment, during treatment, post-assessment and four follow-ups). Table 2 shows the measures used in each phase of the study.

**Table 2:** Measures administration during the different phases of the trial.

Outcome	Eligibility	Pre	Baseline	Treatment	Post	Follow-ups
ADIS-5	X					
Sociodemographic	X					
COVID-19 symptoms	X					
OASIS		X	X	X	X	X
ODSIS		X	X	X	X	X
MEDI		X			X	X
EuroQol-5D		X			X	X
DERS		X			X	X
DTS		X			X	X
CSQ-8					X	
UP-satisfaction					X	

Pre, pre-assessment; Post, post-assessment; ADIS, anxiety and related disorders interview schedule for DSM-5; OASIS, overall anxiety severity and impairment scale; ODSIS, overall depression severity and impairment scale; MEDI, the multidimensional emotional disorder inventory; EuroQol-5D, health-related quality of life; DERS, difficulties in emotion regulation scale; DTS, distress tolerance scale; CSQ-8: client satisfaction questionnaire; UP: Unified Protocol.

#### 2.4.1. Primary outcomes

- Sociodemographic: sex, age, place of residence, marital status, employment status, healthy habits (i.e., smoking, alcohol, physical activity, diet), SARS CoV 2 vaccination. Sociodemographic variables will be evaluated to characterize the sample.
- COVID-19 symptoms: symptoms of post COVID-19 condition referred by the patient according to 10 categories: General, respiratory, gastrointestinal, musculoskeletal, cutaneous, otolaryngological, neurological, cardiovascular, psychological and other symptoms. COVID-19 characteristics will be assessed to describe the sample.
- Overall Anxiety Severity and Impairment Scale (OASIS; Norman et al., 2006; Osma et al., 2021): it consists of 5 items assessing frequency of anxiety symptoms, severity of anxiety symptoms, avoidance and interference. Higher scores indicate more severe anxiety symptoms. Good internal consistency was found in the Spanish version of this instrument (α = 0.87; Osma et al., 2021).
- Overall Depression Severity and Impairment Scale (ODSIS; Bentley et al., 2014; Osma et al., 2021): it comprises 5 items assessing frequency of depressive symptoms, severity of depressive symptoms, anhedonia and interference. Higher scores represent more severe depressive symptoms. Excellent internal consistency was found in the Spanish version of this instrument (*α* = 0.94; Osma et al., 2021).
- Anxiety and Related Disorders Interview Schedule for DSM-5 (ADIS-5; Brown and Barlow, 2014): it is a structured clinical interview to diagnose current anxiety, mood and related disorders (i.e., obsessive-compulsive, trauma, somatic symptoms, substance use) according to the Diagnostic and Statistical Manual of Mental Disorders criteria (5th edition, DSM-5; American Psychiatric Association, 2013). Different editions of the ADIS have shown good reliability with good-to-excellent interrater agreement for DSM disorders (Brown and Barlow, 2014). In this trial, this interview will be used to assess eligibility criteria to participate in the trial.
- Adjustment disorders: adjustment disorders are usually accompanied by medical illness (American Psychiatric Association, 2013). Given the high prevalence of adjustment disorders in the context of the COVID-19 pandemic, and specially in people who experience COVID-19 stressful situations (Ben-Ezra et al., 2021), participants in this trial will be assessed for the presence of adjustment disorders according to DSM-5 criteria (American Psychiatric Association, 2013).

#### 2.4.2. Secondary outcomes

- The Multidimensional Emotional Disorder Inventory (MEDI; Rosellini and Brown, 2019; Osma et al., 2021): it is composed of 49 items that allow the assessment of nine dimensions of the EDs to obtain a transdiagnostic profile. Proposed dimensions include: neurotic temperament, positive temperament, depressed mood, autonomic arousal, somatic anxiety, social anxiety, intrusive cognitions, traumatic re-experiencing and avoidance. Excellent internal consistency was found in the Spanish validation (0.74 ≥ *α* ≤ 0.92; Osma et al., 2021).
- Health-related quality of life (EuroQol-5D; Brooks, 1996; Herdman et al., 2001): it has four different components. In this study, two components were used to collect information about health-related quality of life. The first one consists of 5 items that assess health state on five different domains (i.e., mobility, self-care, usual activities, pain/discomfort and anxiety/depression). The second component evaluates subjective health state on a thermometer (0–100). Higher scores indicate higher quality of life. This instrument has shown good convergent and construct validity in the Spanish version (see Badia et al., 1998)
- Difficulties in Emotion Regulation Scale (DERS; Gratz and Roemer, 2004; Hervás and Jódar, 2008): it is composed of 28 items assessing difficulties in the regulation of emotions when facing distress. It includes 5 dimensions: inattention, confusion, rejection, interference and lack of control over emotions. Higher scores represent more difficulties in the regulation of emotions. Good internal consistency was found in the Spanish version of this instrument (*α* = 0.74; Hervás and Jódar, 2008)
- Distress Tolerance Scale (DTS; Simons and Gaher, 2005; Sandín et al., 2017): it consists of 15 items which assess distressful psychological states. It can be divided in four subscales: perceived ability to tolerate emotional distress, subjective appraisal of distress, attention absorber by negative emotions and regulation efforts to alleviate distress. Lower scores indicate greater ability to tolerate distress. Good internal consistency was found in the Spanish validation (0.83 ≥ *α* ≤ 0.89; Sandín et al., 2017).
- Adaptation of Client Satisfaction Questionnaire (CSQ-8; Larsen et al., 1979): the original version of the CSQ-8 comprises 8 items which assess general satisfaction with services. In our adaptation, 6 of these 8 items were maintained: perceived quality, meeting expectations, recommendation to family or friends, perceived utility of the learned techniques, general satisfaction with the intervention and desire to participate in this kind of intervention in the future. An additional question was included in order to assess discomfort generated by the intervention. The Likert response scale was changed from 4 points (0 = “No, definitely no” – 4 = “Yes, definitely”) to 11-points Likert scale (0 = “Bad/nothing” – 10 = “Excellent/very much”). Finally, to explore the qualitative opinion of participants, five open questions were added: (1) Would you like to include other content that was not addressed in this psychological program? (2) Do you think there is any unnecessary content in this program that you would remove? (3) Do you feel the program duration (8 sessions of 1 h each) is sufficient? (4) How satisfied are you with the format of the program (online and individual)? (5) Please use this space to express any concerns you may have with regard to the program. The original version of the CSQ-8 showed excellent internal reliability (*α* = 0.92; Larsen et al., 1979).
- Evaluation questionnaire of the UP modules: an *ad hoc* questionnaire with 7 items was developed to assess patients’ satisfaction with the psychological programs received. The first question assesses the general perceived utility of the program to improve emotion regulation skills. The remaining 6 questions assess the perceived utility of the program to improve the six abilities that are trained during the UP psychological intervention. Each item is answered using a scale from 0 = “Nothing” to 10 = “Very much,” higher scores indicate greater satisfaction with the UP.
- Baseline assessment: OASIS and ODSIS will be administered daily during baseline and weekly during the treatment phase as a measure of change. Temporality of questions will be changed during baseline to assess anxiety and depressive symptoms during the previous day instead of “during the last week.”

### 2.5. Intervention

All participants who meet the inclusion criteria and consent to participate will receive a psychological intervention based on the “Unified Protocol for the Transdiagnostic Treatment of Emotional Disorders” by Barlow et al. (2018). The UP is a 8 module treatment designed to be applied originally in 12 to 16 individual sessions (Farchione et al., 2021). As the UP developers mentioned, clinicians can be flexible in the number of sessions that are needed to complete each module and also in the order in which modules are presented when applying the UP (Barlow and Farchione, 2017). Thus, in our study the intervention will consist of 8 online individual sessions with a 1 h duration conducted through Google Meet video-calls. As shown in Table 3, five specific psychological abilities (i.e., awareness of emotions, cognitive flexibility, emotion-driven behaviors, exposure to physical sensations and emotional expositions) will be distributed over 8 sessions.

**Table 3:** Sessions and contents of the Unified Protocol-based psychological intervention for patients with post COVID-19 condition.

Session title	Contents and exercises
1. Setting goals and maintaining motivation.	This session is focused on setting general goals to be developed during the intervention as well as the steps to achieve these goals. Exercise – decisional balance: a decisional schedule is worked out with the patient in order to analyse benefit/inconvenience of change and benefit/inconvenience of remaining unchanged. The aim of this exercise is to motivate the patient to move toward the change.
2. Understanding emotions.	In this session, patients learn about the adaptive function of emotions and how to analyse their emotional responses. Exercise – ARC: patients register their emotional responses (Antecedent - Response – Consequences). The aim of this exercise is that patients learn how to identify their emotional responses and how thoughts, physical sensations and behaviors are interrelated.
3. Mindful emotion awareness.	The third session aims to teach how to be in the present without judging the emotional experience. Exercise - Meditation: three different exercises of increasing difficult are proposed (guided meditation, emotion induction and anchoring themselves in the present). The objective of these exercises is for patients to observe their emotional responses in the present and without judgment.
4. Cognitive flexibility.	This session focuses on learning the relationship between thoughts and emotions, learning about automatic irrational thoughts and practicing the cognitive flexibility. Exercise – Cognitions: identification of thoughts and practicing cognitive flexibility. Exercise – Problem solving: identifying problematic situations and proposing rational alternatives. These exercises aim to observe thoughts and analyse whether they are describing the reality and help the patient to achieve their goals (if the patient reports an objective problem, problem-solving should be used) or whether they are irrational thoughts (patients should interpret them in a more flexible way, considering other possibilities).
5. Countering emotional behaviors.	During this session, patients learn the concept of emotionally-driven behaviors and the different kinds of avoidance behaviors. Exercise - Opposite behaviors: this exercise aims to help patients to identify avoidance behaviors and propose an alternative or opposite behavior.
6. Emotional expositions (I).	The sixth session aims to instruct patients on the relationship between body sensations and emotional responses, to encourage patients to face internal and external stimuli that cause distress and to learn how to tolerate them progressively. Exercise – Emotional expositions I: the objective of this exercise is for patients to identify physical unpleasant sensations that cause discomfort. Then, they will train their ability to tolerate them by doing exercises that simulate the body sensations they have identified (i.e., running may increase heart rate).
7. Emotional expositions (II).	In this session, participants continue with the exercises of the previous session through a hierarchy of exposition. Exercise – Emotional expositions II: the aim of this exercise is for patients to identify unpleasant intense emotions in order to design their own hierarchy of exposure to these emotions based on fear and avoidance indicators. The final objective is for patient to learn how to progressively tolerate their emotions.
8. Recognizing accomplishments and looking to the future.	The last session is focused on summarizing the techniques learned during the program and the progress in the management of emotions. Patients also identify future hard situations to propose an adaptive way to overcome them. Exercise – looking to the future: the aim of this exercise is to plan the abilities that need to be reinforced. Patients propose the techniques that will help them in possible difficult situations in the future and propose short-term and long-term objectives to maintain all the progress achieved during therapy.

All sessions will follow the same schedule. New concepts and abilities will be introduced at the beginning of each session. Then, participants will answer true/false questions to ensure they have understood the main ideas of the session and any doubts will be resolved. After the questions, participants will be instructed on how to do the exercises proposed and they will be encouraged to practice at home until the next session. Once the session has finished, participants will receive a manual by e-mail with the main information of the session, the exercises and the corresponding registers.

### 2.6. Data analysis

The Statistical Package for the Social Sciences (SPSS) version 22.0 for windows (IBM Corp, 2013) will be used to conduct the statistical analysis.

First, normal distribution test will be conducted to explore whether the data obtained followed a normal distribution. According to the distribution of the sample, we will use parametric or non-parametric analyses. Then, descriptive data (mean, standard deviation, and proportions) of all study variables will be reported in order to characterize the sample (i.e., sociodemographic and psychosocial status). Missing-value analysis and the Little Missing Completely At Random test (MCAR) will be used to check whether missing values are randomly presented. Last observation carried forward (LOCF) will be used in this case (Jakobsen et al., 2017).

Analysis of Variance (ANOVA), or its equivalent non-parametric Friedman test, will serve to test whether there are differences between the three baseline conditions at pre-assessment to ensure that randomization resulted in comparable groups. Repeated measures ANOVA will help to determine if between-group (differences in outcome variables between the three baseline conditions at different assessment points) and within group differences (differences between the pre-treatment and the remaining assessments in the outcome variables for each baseline condition) are present. Additionally, effect sizes (Cohen’s *d*) will be calculated for both between and within group analyses. Effect sizes will be interpreted as small (*d* ≈ 0.2), medium (*d* ≈ 0.5) or large (*d* ≈ 0.8).

As recommended for n-of-1 trials, visual analysis of the data by graphs will also be used to determine whether the psychological outcomes improve as a consequence of intervention contents. By doing so, we will establish the baseline pattern on OASIS and ODSIS scales and compare it with the data within the following treatment phase and also with results obtained during the follow-up assessments. The statistical Software R version 4.1.0 (R Core Team, 2021) will be used for the graphical representation of slopes.

Adherence to the intervention will be calculated (proportion of attendance to sessions compared with programed ones) as a measure of programs acceptability. With the same purpose, we will conduct qualitative analyses of reports provided in open questions regarding the satisfaction with the program. Content analysis will be performed with the MAXQDA program (Kuckartz and Rädiker, 2019).

## 3. Discussion

During the last years, the entire globe has faced an extraordinary health emergency due to the COVID-19 pandemic. During this 2-year period, millions of individuals have suffered the impact of the pandemic itself and its negative consequences (WHO, 2022a). Some people have developed adequate emotion regulation skills and have adapted to the challenges imposed by the pandemic. However, there is a sizeable percentage of individuals that have found it challenging to regulate their emotions and have developed EDs (Liu et al., 2021). Recent studies have revealed that psychological interventions could be useful in the management of EDs in the context of the pandemic (Yue et al., 2020; Bertuzzi et al., 2021; Rahmati and Khalili, 2022). Unfortunately, with current psychological symptoms-focused interventions, it is difficult to address comorbid EDs. Additionally, an extreme vulnerable population, namely patients suffering long COVID-19 conditions, are generally excluded in these studies so we cannot determine whether psychological interventions could alleviate psychological suffering in this population.

In the present protocol, we describe the procedures for a multiple baseline n-of-1 trial which aims to assess the clinical utility and acceptability of the UP for the transdiagnostic treatment of EDs in a sample of patients with long COVID-19. We hypothesized that the UP will be effective in the improvement of EDs or symptoms and this intervention will be well-accepted by long COVID-19 participants. Specifically, we expect that the UP intervention will help patients to understand the adaptive function of emotions, adopt a non-judgmental attitude toward their unpleasant emotions, practice the cognitive flexibility, overcome avoidance behaviors, and increase their tolerance to physical sensations and unpleasant emotions. As previous literature has shown (Khakpoor et al., 2019; Sakiris and Berle, 2019; Cassiello-Robbins et al., 2020; Carlucci et al., 2021; Schaeuffele et al., 2022; Longley and Gleiser, 2023; Peris-Baquero et al., 2023), we believe that training in these skills may help patients to improve their emotion regulation skills, neuroticism scores and emotion avoidance (transdiagnostic factors) and it may result in a reduction on anxiety and depression symptomatology and an improvement on quality of life. If our hypothesis is confirmed, we would demonstrate that a brief transdiagnostic psychological intervention could be implemented in healthcare systems to alleviate psychological suffering in long COVID-19 patients. Also importantly, results from this trial may support results from previous studies that postulated emotion regulation deficits are relevant in the development and maintenance of EDs (Linehan, 1993; Gratz, 2003). Different scientific studies have highlighted the relevance of emotion regulation in challenging periods as the COVID-19 crisis (Waterschoot et al., 2022) and have claimed the need for delivering interventions that help to develop adaptive emotion regulation strategies (Zhao et al., 2021). Our study could be a first step in this direction.

Furthermore, results from this study may help to highlight the relevance of maintaining and increasing participants’ quality of life and the usefulness of an adapted UP intervention for this purpose. During the first phases of the COVID-19 crisis, medical efforts were concentrated on stopping the spread of the illness and on improving the survival rate. Two years later, the focus needs to be maintained on contingency measures and severity of symptoms reduction, but we cannot forget that health is not only absence of disease (WHO, 2020) and, consequently, we should also promote good levels of quality of life.

While acknowledging these relevant contributions, the present study has certainly some limitations. First, blinding professionals and participants to baseline conditions is not possible. The same psychological intervention will be applied to all participants irrespective of their baseline condition so we expect that unblended allocation will not affect the trial outcomes. Second, it is important to note that some study characteristics (i.e., eligibility criteria) may limit the generalization of our results to all long COVID-19 populations. In this sense, exclusion criteria may impede some long COVID-19 participants the possibility of participating in this study. Participants currently undergoing psychological or pharmacological treatment cannot participate in this trial. Co-prescribing is one of the most common exclusion criteria in clinical trials for physical conditions (He et al., 2020). In our case, concurrent pharmacological or psychological treatment could interfere with the psychological intervention provided in this trial and it will make it difficult to interpret the results. Finally, participants with low Internet access (i.e., not having Internet connexion or not able to engage in video-calls) will not participate in the intervention as it will be the main channel of communication. Given that in 2022 it was estimated that 93% of Europeans have Internet access (Eurostat, 2017), we expect the impact of this exclusion criteria to be minimal.

Despite the aforementioned limitations, to the best of our knowledge, this is the first study that tests the clinical utility and acceptability of a transdiagnostic psychological intervention provided to patients diagnosed with post COVID-19 condition. Our findings will serve to provide valuable information that may change the model of care provided to long COVID-19 patients from mainly physical rehabilitation to integrating a multidisciplinary model of care that considers the inclusion of psychological components. Thanks to the outcomes of this trial, healthcare systems would provide an evidence-based intervention to patients who present long COVID-19 conditions and comorbid EDs.

## Ethics statement

Approval from the Research Ethics Committee of the Autonomous Community of Aragón (CEICA) was obtained to conduct this trial. All changes to this original study protocol (current protocol version number 6; December 15th, 2022) will be communicated to the aforementioned Ethics Committee for their approval and will be shown in clinicaltrials.gov.

## Author contributions

JO and VM-B: conceptualization and methodology. VM-B, LM-G, and ÓP-B: writing - original draft preparation. JO and EC-B: writing – review and editing. EC-B: supervision and project administration. All authors contributed to the article and approved the submitted version.
