# Supplementary material for: Unified Protocol for the transdiagnostic treatment of emotional disorders in people with post COVID-19 condition: study protocol for a multiple baseline n-of-1 trial
Source: Front Psychol. 2023 Oct 18;14:1160692. doi: 10.3389/fpsyg.2023.1160692 (PMC10618554; doi:10.3389/fpsyg.2023.1160692)
Supplement: Supplementary file 1 [file Data_Sheet_1.pdf]

Supplementary Material

Unified Protocol for the Transdiagnostic Treatment of Emotional Disorders in people with Post COVID-19 condition: study protocol for a multiple baseline n-of-1 trial

Verónica Martínez-Borba[1], Laura Martínez-García[1,2], Oscar Peris-Baquero[1,2], Jorge Osma[1,2*], Esther del Corral-Beamonte[3]

*** Correspondence:** Jorge J. Osma López: osma@unizar.es

Supplementary material A. Spirit extension for N-of-1 trials (SPENT) Checklist.

SECTION / TOPIC	ITEM NO.	SPIRIT 2013	ITEM NO.	SPENT 2019	ON PAGE
**SECTION 1: ADMINISTRATIVE DATA**					
Title	1	Descriptive title identifying the study design, population, interventions, and, if applicable, trial acronym.	1a	Descriptive title, including "N-of-1 trial" and “protocol”. ***For series:*** Descriptive title, including "a series of N-of-1 trials" and “protocol”.	Title page
		—	1b	For specific guidance on abstracts, see SPENT Guidance for Abstracts. (Appendix B)	Suppl. B
Trial Registration	2a	Trial identifier and registry name. If not yet registered, name of intended registry.	2a	(no change)	Title page, 3
	2b	All items from the World Health Organization Trial Registration Data Set. (WHOTRDS)	2b	(no change)	clinicaltrials.gov
Protocol version	3	Date and version identifier.	3	(no change)	9
Funding	4	Sources and types of financial, material, and other support.	4	(no change)	11
Roles and responsibilities	5a	Names, affiliations, and roles of protocol contributors.	5a	(no change)	11
	5b	Name and contact information for the trial sponsor.	5b	(no change)	11
	5c	Role of study sponsor and funders, if any, in study design; collection, management, analysis, and interpretation of data; writing of the report; and the decision to submit the report for publication, including whether they will have ultimate authority over any of these activities.	5c	(no change)	11
	5d	Composition, roles, and responsibilities of the coordinating center, steering committee, end point adjudication committee, data management team, and other individuals or groups overseeing the trial, if applicable. (see Item 21a for DMC)	5d	(no change)	4
**SECTION 2: INTRODUCTION**					
Background and rationale	6a	Description of research question and justification for undertaking the trial, including summary of relevant studies (published and unpublished) examining benefits and harms for each intervention.	6a	Description of research question and justification for undertaking the trial, including summary of relevant studies (published and unpublished) examining benefits and harms for each intervention, and rationale for using N-of-1.	2-3

	6b	Explanation for choice of comparators.	6b	(no change, SPENT commentary)	NA
Objectives	7	Specific objectives or hypotheses.	7	(no change)	3
Trial design	8	Description of trial design, including type of trial (e.g., parallel group, crossover, factorial, single group), allocation ratio, and framework (e.g., superiority, equivalence, non-inferiority, exploratory).	8	Description of the trial design, including N-of-1 trial or series of trials, and framework (e.g., superiority, equivalence, non-inferiority, exploratory). ***In addition for series:*** Explanation of the series design including whether the design will be tailored to each participant.	3-5
**SECTION 3: METHODS**					
Participants, interventions, and outcomes					
Study Setting	9	Description of study settings (e.g., community clinic, academic hospital) and list of countries where data will be collected. Reference to where list of study sites can be obtained.	9	(no change)	4
Eligibility criteria	10	Inclusion and exclusion criteria for participants. If applicable, eligibility criteria for study centers and individuals who will perform the interventions (e.g., surgeons, psychotherapists).	10	(in addition) Diagnosis/disorder, diagnostic criteria, co-morbid conditions and concurrent therapies. ***For series:*** Same as SPIRIT item 10.	4 and table 1
Interventions	11a	Interventions for each group with sufficient detail to allow replication, including how and when they will be administered.	11a	Intervention(s) for each period with sufficient detail to allow replication, including how and when they will be administered, planned number of periods, and duration of each period (including run-in and washout, if applicable).<br>***In addition for series:*** How the design will be tailored to each participant, if applicable.	7 and table 3
	11b	Criteria for discontinuing or modifying allocated interventions for a given trial participant (e.g., drug dose change in response to harms, participant request, or improving/worsening disease).	11b	(no change, SPENT commentary)	4-5
	11c	Strategies to improve adherence to intervention protocols, and any procedures for monitoring adherence (e.g., drug tablet return, laboratory tests).	11c	(no change)	5
	11d	Relevant concomitant care and interventions that are permitted or prohibited during the trial.	11d	(no change)	4, 9 and table 1
Outcomes	12	Primary, secondary, and other outcomes, including the specific measurement variable (e.g., systolic blood pressure), analysis metric (e.g., change from baseline, final value, time to	12	(no change)	6, 7 and table 2

		event), method of aggregation (e.g., median, proportion), and time point for each outcome. Explanation of the clinical relevance of chosen efficacy and harm outcomes is strongly recommended.			
Participant timeline	13	Time schedule of enrollment, interventions (including any run-ins and washouts), assessments, and visits for participants. A schematic diagram is highly recommended (Figure).	13	(no change)	4-5 and Figure 1
Sample size	14	Estimated number of participants needed to achieve study objectives and how it was determined, including clinical and statistical assumptions supporting any sample size calculations.	14	Estimated number of intervention periods and measurements/observations needed to achieve study objectives within an individual N-of-1 trial. ***In addition for series:*** Estimated number of participants needed to achieve study objectives. How these numbers were determined, including clinical and statistical assumptions supporting any sample size calculations.	4
Recruitment	15	Strategies for achieving adequate participant enrollment to reach target sample size.	15	***For series:*** Strategies for achieving adequate participant enrollment to reach target sample size.	4
Assignment of interventions (for controlled trials)					
Allocation					4-5
sequence generation	16a	Method of generating the allocation sequence (e.g., computer-generated random numbers), andlist of any factors for stratification. To reduce predictability of a random sequence, details of any planned restriction (e.g., blocking) should be provided in a separate document that is unavailable to those who enroll participants or assign interventions.	16a	Method of generating the allocation sequence (e.g., computer-generated random numbers), and list of any factors for stratification. To reduce predictability, details of any restrictions (e.g., pairs, blocking) should be provided in a separate document that is unavailable to those who enrol participants or assign interventions. ***In addition for series:*** List of any factors for stratification.	
concealment mechanism	16b	Mechanism of implementing the allocation sequence (e.g., central telephone; sequentially numbered, opaque, sealed envelopes), describing any steps to conceal the sequence until interventions are assigned.	16b	(no change, SPENT commentary)	4-5
implementation	16c	Who will generate the allocation sequence, who will enroll participants, and who will assign participants to interventions.	16c	(no change)	4-5
Blinding (masking)	17a	Who will be blinded after assignment to interventions (e.g., trial participants, care providers, outcome assessors, data analysts), and how.	17a	(no change)	4-5, 9

	17b	If blinded, circumstances under which unblinding is permissible, and procedure for revealing a participant's allocated intervention during the trial.	17b	(no change)	4-5, 9
Data collection, management, and analysis					
Data collection methods	18a	Plans for assessment and collection of outcome, baseline, and other trial data, including any related processes to promote data quality (e.g., duplicate measurements, training of assessors) and a description of study instruments (e.g., questionnaires, laboratory tests) along with their reliability and validity, if known. Reference to where data collection forms can be found, if not in the protocol.	18a	(no change)	5-7
	18b	Plans to promote participant retention and complete follow-up, including list of any outcome data to be collected for participants who discontinue or deviate from intervention protocols.	18b	(no change)	5
Data management	19	Plans for data entry, coding, security, and storage, including any related processes to promote data quality (e.g., double data entry; range checks for data values). Reference to where details of data management procedures can be found, if not in the protocol.	19	(no change)	9-10
Statistical methods	20a	Statistical methods for analyzing primary and secondary outcomes. Reference to where other details of the statistical analysis plan can be found, if not in the protocol.	20a1	Statistical methods for analyzing primary and secondary outcomes for each individual. Reference to where other details of the statistical analysis plan can be found, if not in the protocol. ***In addition for series:*** if planned, proposed methods of quantitative synthesis of individual trial data, and how heterogeneity between participants will be assessed	7-8
			20a2	Statistical methods to account for correlation introduced by the repeated measures and crossover design of N-of-1 studies.	7-8
	20b	Methods for any additional analyses (e.g., subgroup and adjusted analyses).	20b	***For series:*** Methods for any additional analyses (e.g., subgroup and adjusted analyses).	7-8
	20c	Definition of analysis population relating to protocol non-adherence (e.g., as-randomized analysis), and any statistical methods to handle	20c	Statistical methods to handle missing data (e.g., multiple imputation, modelling). ***In addition for series:*** Definition of analysis population relating	8

		missing data (e.g., multiple imputation).		to protocol non-adherence (e.g., as-randomized analysis).	
**Monitoring**					
Data monitoring	21a	Composition of Data Monitoring Committee (DMC); summary of its role and reporting structure; statement of whether it is independent from the sponsor and competing interests; and reference to where further details about its charter can be found, if not in the protocol. Alternatively, an explanation of why a DMC is not needed.	21a	(no change, SPENT commentary)	10
	21b	Description of any interim analyses and stopping guidelines, including who will have access to these interim results and make the final decision to terminate the trial.	21b	(no change, SPENT commentary)	10
Harms	22	Plans for collecting, assessing, reporting, and managing solicited and spontaneously reported adverse events and other unintended effects of trial interventions or trial conduct.	22	(no change, SPENT commentary)	5, 10
Auditing	23	Frequency and procedures for auditing trial conduct, if any, and whether the process will be independent from investigators and the sponsor.	23	(no change)	10
**SECTION 4: ETHICS & DISSEMINATION**					
Research ethics approval	24	Plans for seeking REC/IRB approval.	24	(no change, SPENT commentary)	9
Protocol amendments	25	Plans for communicating important protocol modifications (e.g., changes to eligibility criteria, outcomes, analyses) to relevant parties (e.g., investigators, RECs/IRBs, trial participants, trial registries, journals, regulators).	25	(no change)	9
Consent or assent	26a	Who will obtain informed consent or assent from potential trial participants or authorized surrogates, and how (see item 32).	26a	(no change)	4 and figure 1
	26b	Additional consent provisions for collection and use of participant data and biological specimens in ancillary studies, if applicable.	26b	(no change)	NA
Confidentiality	27	How personal information about potential and enrolled participants will be collected, shared,	27	(no change, SPENT commentary)	9-10

		and maintained in order to protect confidentiality before, during, and after the trial.			
Declaration of interests	28	Financial and other competing interests for principal investigators for the overall trial and each study site.	28	(no change)	10
Access to data	29	Statement of who will have access to the final trial data set, and disclosure of contractual agreements that limit such access for investigators.	29	(no change)	10
Ancillary and post-trial care	30	Provision, if any, for ancillary and post-trial care, and for compensation to those who suffer harm from trial participation.	30	(no change)	4-5
Dissemination policy	31a	Plans for investigators and sponsor to communicate trial results to participants, health care professionals, the public, and other relevant groups (e.g., via publication, reporting in results databases, or other data-sharing arrangements), including any publication restrictions.	31a	Plans for investigators to communicate each individual's results to the participant. Plans for investigators and sponsor to communicate trial results to participants, health care professionals, the public, and other relevant groups (e.g., via publication, reporting in results databases, or other data-sharing arrangements), including any publication restrictions.	10
	31b	Authorship eligibility guidelines and any intended use of professional writers.	31b	(no change)	9-10
	31c	Plans, if any, for granting public access to the full protocol, participant-level data set, and statistical code.	31c	(no change)	9-10
**SECTION 5: APPENDICES**					
Informed consent materials	32	Model consent form and other related documentation given to participants and authorized surrogates.	32	(no change)	Available upon reasonable request
Biological specimens	33	Plans for collection, laboratory evaluation, and storage of biological specimens for genetic or molecular analysis in the current trial and for future use in ancillary studies, if applicable.	33	(no change)	NA

Supplementary material B: SPENT abstracts checklist

Item	Extension for N-of-1 protocol abstracts	Included on abstract?
Title	Identification as a protocol of an n-of-1 trial or series of n-of-1 trials in the title.*	Yes
Context and rationale	Description of research question and justification for undertaking the trial; rationale for using n-of-1.	Yes
Objectives	Specific objective or hypothesis.	Yes
Methods		
Settings	Description of study settings and list of countries where data will be collected.	Yes
Participants	For individual trials: clinical condition under study. For series: eligibility criteria for participants.*†	Yes
Sample size	For series: estimated sample size.†	Yes
Trial design	Description of trial design, including number of periods, and period duration.*	Yes
Interventions	Interventions intended for each period.*	Yes
Outcomes and data collection	Clearly defined primary outcome, including the specific measurement variable; secondary outcomes if relevant to participant/trial goals. Explanation of the clinical relevance of chosen outcomes is strongly recommended.*	Yes
Randomisation	Sequence blocking and generation.	Yes
Blinding (masking)	Whether the participants, care givers, and those assessing and analysing the outcomes will be blinded after assignment tointerventions.	Yes
Analysis	For the outcomes listed, planned analyses.	Yes
Discussion	A brief summary and potential implications of the trial, including any ethics review information and dissemination plans.	Yes
Trial registration	Statement of registry and number, protocol version number and date.	Yes

SPIRIT=standard protocol items: recommendations for interventional trials; CENT=CONSORT (consolidated standards of reporting trials) extension for n-of-1 trials.
*Based on CENT wording.

†The term "for series" refers to issues not applicable to individual participants or trials.

Supplementary material C. Inclusion and exclusion criteria to participate in the nutritional and rehabilitation intervention for patients with post COVID-19 (ARACOV project).

**Inclusion**	**Exclusion**
Documented COVID-19 infection (i.e., PCR, Ag test or serology).	Patients with severe neurological diseases.
Presenting Post COVID-19 condition (i.e., persistence of COVID-19 symptoms beyond 12 weeks after the initial infection).	Presenting respiratory insufficiency (oxygen saturation <90% or respiratory frequency ≥ 30.
Over 18 years old.	Presenting rheumatic pathology or acute musculoskeletal injury.
Presenting fatigue scores ≥ 4 points on FSS.	Not having Internet access.
Independent ambulation, even with the use of technical aids.	Patients with chronic fatigue.
Signing informed consent	Patients with insufficient understanding of Spanish.
	Patients with allergies to fish, shellfish, crustaceans or to any of the components or the supplement.
	Patients currently participating, or who had participated in a clinical trial in the previous 4 weeks before the eligibility assessment.
	Patients who had receive an immunosuppressant and/or corticosteroids during the past weeks.
